# Patient-Specific Devices for Surgical Treatment of Mandibular Condylar Head Fractures: Case Series of Early Experiences and Pitfalls

**DOI:** 10.1007/s12663-025-02799-4

**Published:** 2025-11-18

**Authors:** Wenko Smolka, Paris Liokatis, Katharina Obermeier, Carl-Peter Cornelius

**Affiliations:** Department of Oral and Maxillofacial Surgery, University of Munich, LMU, Lindwurmstr. 2a, 80337 Munich, Germany

**Keywords:** Mandible, Condylar head fracture, Open reduction, Internal fixation, Patient specific devices

## Abstract

Open reduction and internal fixation (ORIF) of condylar head fractures (CHF) can be challenging. Virtual planning and CAD/CAM-based patient-specific devices (PSD) offer to enhance the accuracy of fragment reduction. We present four CHF cases demonstrating the strengths and shortcomings of different PSD designs. From this still limited experience, some fundamental recommendations evolved: • The PSD design should permit adequate visualization of the fracture line to ensure anatomical fracture reduction. • The PSD for reposition and prefixation of the medial fragment should incorporate an abundance of screw holes, particularly in its lateral section, since this part is most easily accessible intraoperatively. • PSDs extended over the dome of the condylar head should be omitted to avoid interferences between fragments, PSD and the adjacent glenoid fossa.

## Introduction

Mandibular condylar head fractures (CHF) can be treated non-surgically or through open reduction and internal fixation (ORIF) [1]. ORIF is the preferred modality when the CHF lines are located within or below the lateral pole zone of the condyle and accompanied by dislocation of the medial fragment, resulting in loss of the vertical ramus height [2]. ORIF has been demonstrated to provide superior morphological and functional outcomes compared to non-surgical therapy of CHF [3–5].

Various techniques for ORIF of CHF were described in the literature including microplates, miniplates, lag screws and positional screws [6–8]. Lateral small-fragment positional screw osteosynthesis (LSFPSO) has shown good functional and stable long-term outcomes [2]. Still, accurate reduction and fixation of the medial bone portions may be demanding above all in major fragmentation [9].

Virtual surgical planning (VSP) and computer-aided design/ computer-aided manufacturing (CAD/CAM) PSDs provide high accuracy for reduction of bone segments or fragments and are current best practice in orthognathic surgery and post-trauma or oncologic reconstruction of the facial skeleton [10–12].

This case series (from 2017 to 2020) reviews PSDs for ORIF of CHF, analyses their advantages and limitations, and suggests design refinements to overcome identified drawbacks.

## Case 1

38-year-old male with bilateral mandibular CHF and a symphyseal fracture resulting from interpersonal violence.

### Clinical Findings

Limited jaw opening, bilateral preauricular swelling, malocclusion, frontal open bite.

### Imaging and Planning

**Fig. 1:**
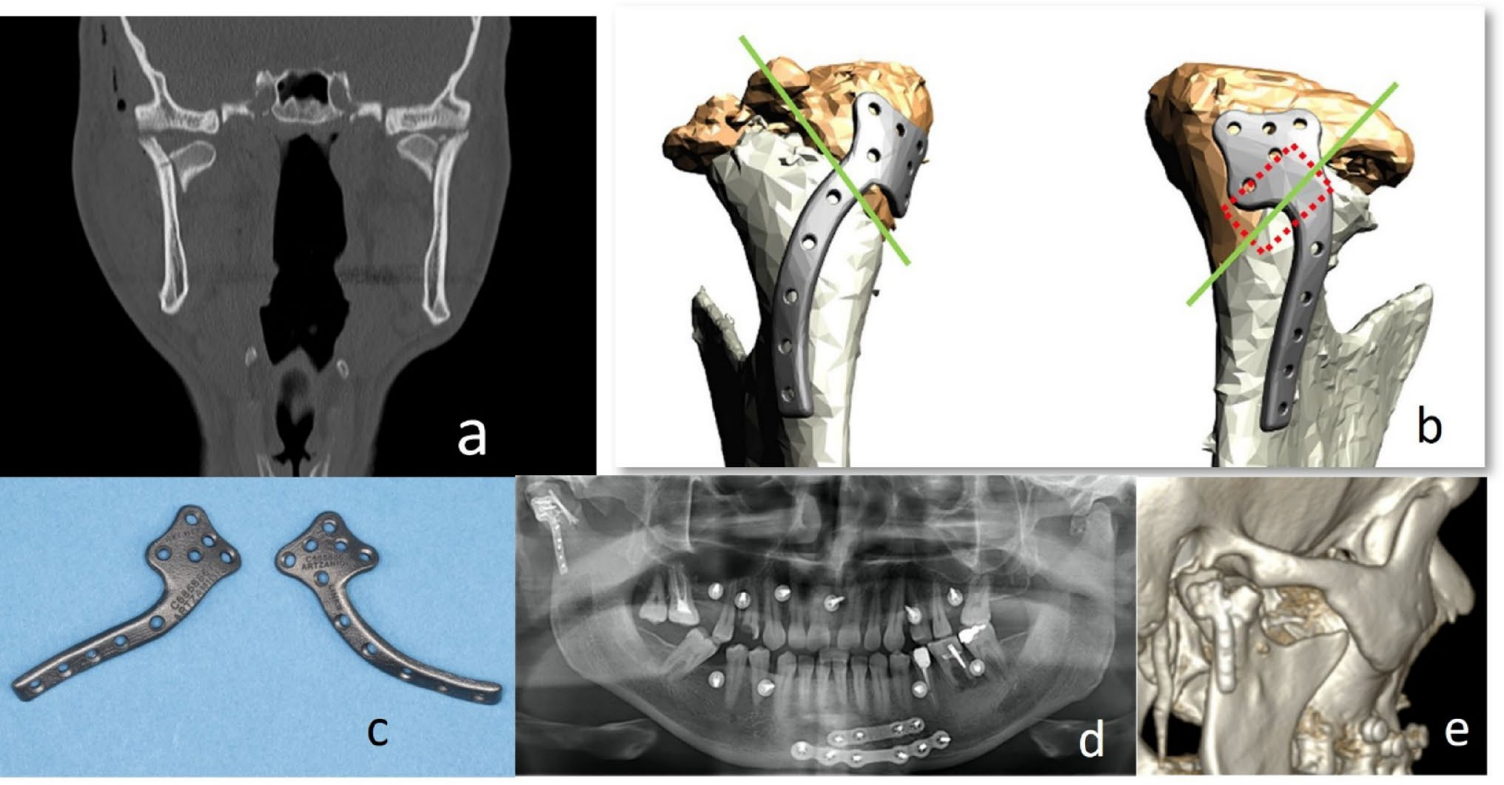
Case 1: Preoperative CT-Scan (coronal plane) – bilateral mandibular CHF: right side – AO type-p without fragmentation and lost vertical apposition; left side – AO type-p with minor fragmentation and lost vertical apposition (**a**). VSP and CAD dorsolateral views of both CHF – green lines indicate the fracture plane; right CHF with fracture line widely covered by the rhomboid PSD shield (red rectangle) (**b**). CAD/CAM PSDs (KLS Martin, Tuttlingen, Germany) (**c**). Postoperative Imaging – Panorex (**d**); 3D CT-Scan – dorsolateral view (**e**)

High-resolution (0.625 mm) Computed Tomography (CT) revealed bilateral CHF, both lateral to the lateral pole zone (AO type-p) **(**Table 1**)** with lost vertical apposition, no fragmentation on the right, and minor fragmentation on the left (Fig. 1).

Interactive VSP with an industrial partner (KLS Martin Group, Tuttlingen, Germany) went for a PSD design to assist anatomical fracture reduction of the medial condylar fragment and prefixation prior to definitive fixation with a two screw LSFPSO. The PSDs were intended for removal at the operation’s conclusion.

### Implant Design

The PSD design featured a solid rhomboid shield in the upper part for reduction and prefixation of the medial fragment, transitioning into a curved lower end below the fracture line for fixation along the lateral surface of the ramus. Multiple holes enabled flexibility for optimal screw placement, accommodating emerging access limitations or compromised bone quality.

### Surgical Procedure

The symphyseal fracture underwent ORIF with two miniplates. The right CHF was exposed through a preauricular approach. The medial condylar fragment, displaced antero-caudo-medially, was reduced by means of sharp hooks and diamond-coated elevators. The placement of the PSD obstructed the fracture line with its rhomboid shield, necessitating a change in strategy. Instead of the PSD a grid plate (1.2 mm screws; Medartis AG, Basel, Switzerland) was used for prefixation of the medial fragment after anatomical reduction. The PSD was then placed onto the plate and mounted (1.2 mm screws) for reinforcement. Two small-fragment screws (1.8 mm, Medartis) were inserted in typical LSFPSO manner. The left-side ORIF with the second PSD was deferred because a reproducible multipoint occlusion had been achieved via reduction of the right condylar head.

### Outcome

Postoperative Panorex confirmed adequate reduction and fixation. Functional treatment with elastics was performed for three weeks.

## Case 2

13-year-old boy with a right mandibular CHF caused by a fall.

### Clinical Findings

Limited jaw opening, contralateral open bite.

### Imaging and Planning

**Fig. 2:**
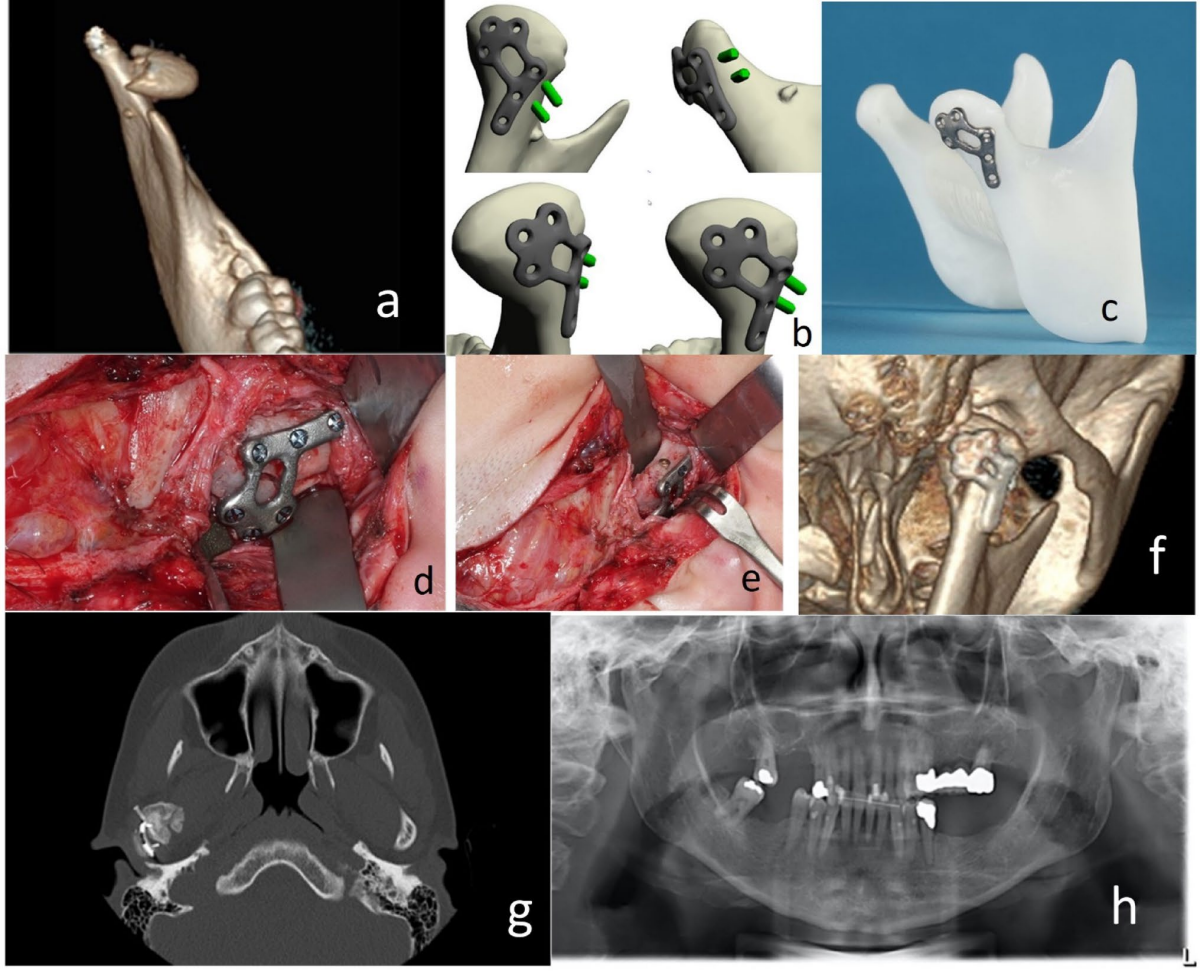
Case 2: Preoperative 3D CT-Scan – right mandibular CHF (**a**). VSP – mirrored condyle with P-shaped PSD. Outer portion of two screws for lateral fixation in green (**b**). CAM – PSD on STL model (**c**). Intraoperatively – fixation of the P-shaped PSD after CHF reduction (**d**). Intraoperatively – head of single 15 mm long small fragment screw in ramus fracture end (**e**). Postoperative 3D CT-Scan – combined PSD and LSFPSO reconstruction (**f**). CT-Scan 7 months postoperatively – PSD with loosening of its fixation screws (**g**)

CT imaging revealed an AO type-p CHF with no fragmentation but lost vertical apposition. The medial fragment exhibited an atypical rectangular shape and was displaced antero-medially and angulated caudally (Fig. 2).

VSP mirrored the contralateral condylar head to define the pre-injury position of the fragment as foundation for the PSD design. This approach was comfortable but ignored the unique shape of the medial fragment and the fracture line’s course, eventually leading to suboptimal PSD fit.

### Implant Design

A P-shaped PSD with three screw holes for dorsal fixation of the medial fragment and additional holes for lateral neck fixation was designed. Angulated insertion points for two positional screws were incorporated. The PSD allowed for visualization of the fracture line; removal at the end of the operation was planned.

### Surgical Procedure

The PSD bridged the fracture. The fragment’s flat configuration allowed LSFPSO with one screw (1.8 mm, length:15 mm; Medartis) only. Consequently, the PSD was left in situ as stand-by to reinforce overall stability.

### Outcome

Postoperative CT confirmed proper reduction and fixation.

Elastics were used for 10 days. At seven months postoperatively, bony consolidation was observed, though screw and plate loosening necessitated hardware removal. Preinjury occlusion was stable, and jaw opening unrestricted.

## Case 3

59-year-old woman after right CHF, initially treated non-surgically.

### Clinical Findings

Malocclusion, right temporomandibular joint pain 16 months post injury.

### Imaging and Planning

**Fig. 3:**
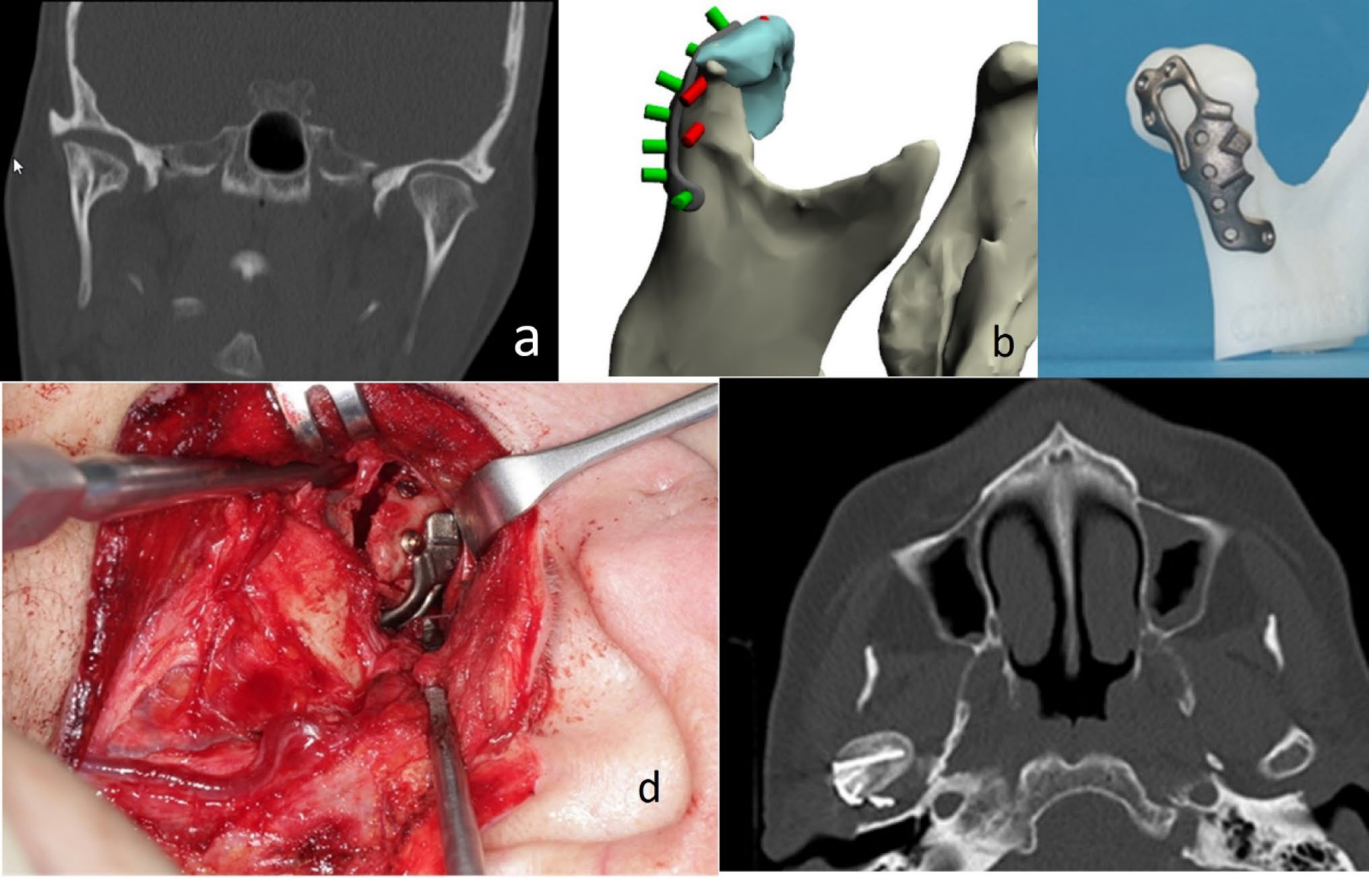
Case 3: Preoperative Panorex – deformity after conservative treatment and bone healing of the right condylar head following a displaced AO type-p CHF due to reduced vertical apposition of the medial fragment (**a**). Preoperative CT-Scan (coronal plane) – loss of vertical ramus height by caudal displacement of the medial fragment (**b**). VSP/CAD – reosteotomy and reduction of the medial condylar head segment (light blue); PSD (dark grey) fixed with micro screws (green) combined with LSFPSO via two screws (red) (**c**). STL-model with PSD (**d**). Intraoperatively – PSD in situ plus the heads of two screws for LSFPSO: one screw head next to the superior indicator notch, second screw head in more antero-superior position than planned (**e**). Postoperative CT-Scan (axial plane) (f)

Imaging revealed deformation of the right condylar head with vertical ramus height loss due to medial fragment displacement (Fig. 3). VSP led to the implementation of a crescent-shaped osteotomy for mobilization and reduction of the medial segment and PSD-assisted fixation. The virtual reduction of the medial fragment produced an osteotomy gap.

### Implant Design

Due to this gap a large-scale, rigid P-shaped PSD was designed with a fenestration to inspect the osteotomy gap, multiple screw holes for bony anchoring and LSFPSO indicators. The upper arm contained 3 screw receiving holes. The lower arm descended on the posterior surface of the condylar process with a curvature below the level of the sigmoid notch to create enough space for five screw holes. The principal load bearing function relied on the LSFPSO. The optimal entry points for two respective screws were indicated by triangular laser-etched notches in the lower arm of the PSD.

### Surgical Procedure

A piezosurgery osteotomy was performed, and the medial condylar segment was repositioned into the glenoid fossa. The PSD was applied uneventfully. Owing to poor bone quality, prefixation of the medial segment with a screw was limited to the uppermost PSD hole. As anticipated an irregular gap remained between the segments. The upper screw for the LSPSO could be inserted guided by the notch indicator. Since the form of the bony gap differed from VSP due to the material reduction during piezosurgical sawing the second screw was inserted free hand antero-superiorly of the indicating notch and angulated in a vertical direction to safely purchase the medial fragment.

### Outcome

X-rays confirmed adequate reduction of the condylar head, proper location in the glenoid fossa and restored vertical ramus height.

Six months postoperatively, the patient developed right-sided preauricular pain and restricted jaw opening. CT-Imaging revealed a contact of the PSD with the glenoid fossa. Hardware removal alleviated symptoms, and further follow-up was unremarkable.

## Case 4

62-year-old man with left mandibular multi-fragmented CHF following a bike accident.

### Clinical Findings

Severe occlusal disturbances, ipsilateral open bite.

### Imaging and Planning

**Fig. 4:**
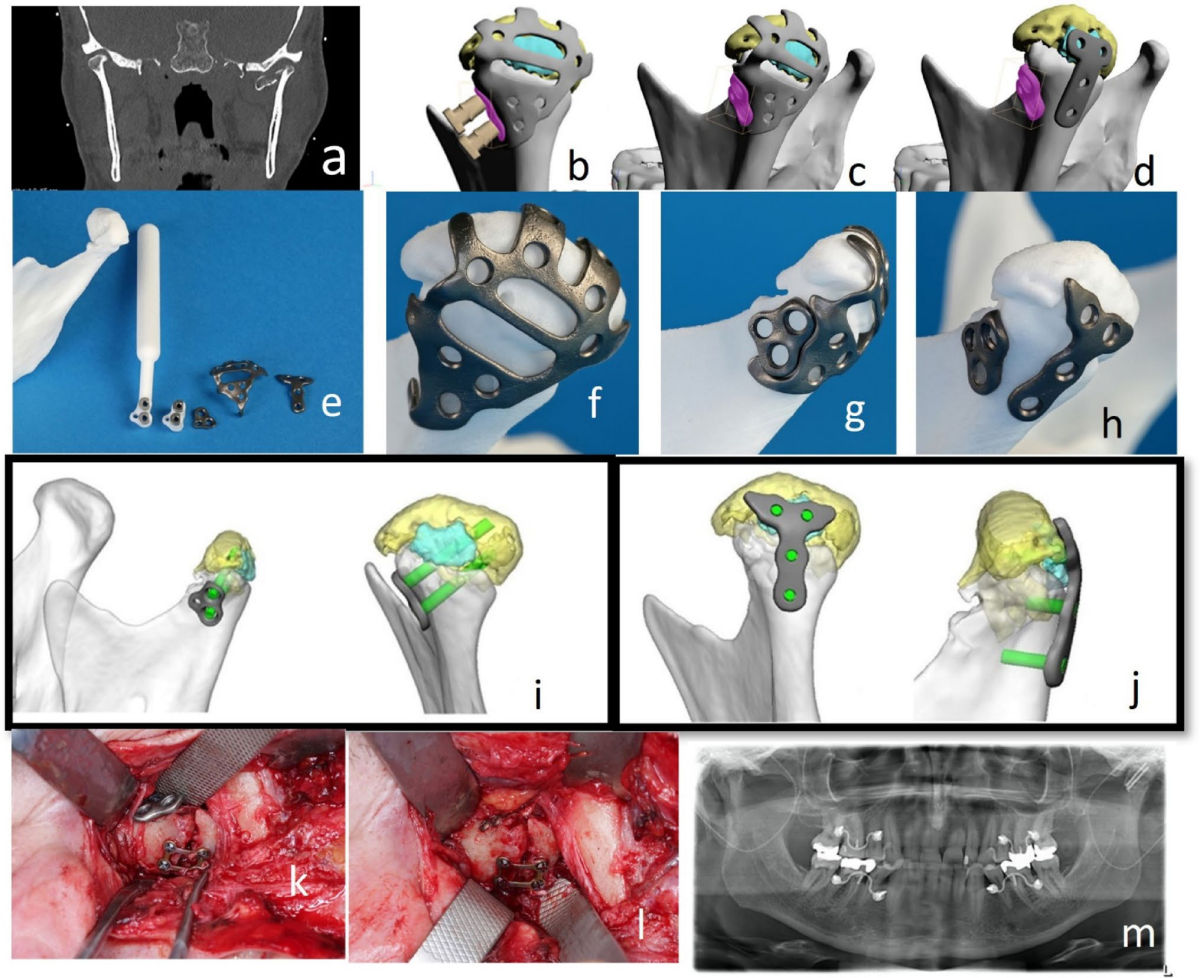
Case 4: Preoperative CT-Scan (representative coronal view) – left mandibular AO type-p CHF with multi-fragmentation (three pieces) and lost vertical apposition (**a**). VSP/CAD – fracture reduction and PSD components (dorsal view) - large medial dome fragment (yellow), SDIMF (cyan). Dome fragment in unison with SDIMF encompassed by upper portion of primary PSD component; bipartite central window in PSD midportion; lower PSD portion surrounds postero-lateral condylar neck and base transition zone; navigation/washer PSD in its docking position; cylindrical drill guides attached (**b**). VSP/CAD – primary PSD component plus navigation/washer PSD in key-lock position (**c**). VSP/CAD – finally remaining PSD components: primary PSD removed, navigation/washer PSD in situ, T- shaped PSD over posterior condylar surface retaining SDIMF (**d**). Overview – STL-model (left condylar process reduced), 2 drill guides, 3 distinct PSDs (from left to right) for reduction/prefixation, navigation/washer function and fragment retention/ reinforcement (**e**). STL-model – dorsal view of reduced CHF with reduction/prefixation PSD component (**f**). Same STL-model – lateral view after tongue-in-groove docking of navigation/washer PSD and reduction/prefixation PSD (**g**). Same STL-model – dorso-lateral view with primary PSD removed, navigation/washer PSD left in place, additional T-shaped retaining/ reinforcement PSD (**h**). VSP – navigation/washer PSD placed under LSFPSO; intramedullary LSFPSO pathways (green) (i). VSP – T-shaped PSD fixated with small diameter screws (green) (j). Intraoperatively – temporary fixation of medial condylar head fragment with conventional grid plate; navigation/washer PSD fastened (k). Same view after removal of the washer and insertion of 2 lateral screws (l). Postoperative Panorex (m)

CT-Scans delineated a left AO type-p CHF with multi-fragmentation (three major fragments), loss of vertical apposition, and anteromedial displacement (Fig. 4).

A three-component multi-purpose PSD was designed. In line with previous cases, one PSD component of the PSD was designated to reduction and prefixation. To address the difficulties in stabilizing the dislocated largest medial fragment alongside smaller pieces, the upper PSD portion was equipped with four finger-like extensions along the entire posterior surface of the condylar head. The middle section of the PSD incorporated a large window for unobstructed visualization of the fracture line and its cranial ramifications. The lower PSD portion encompassed the neck region, reaching down to the level of the deepest point of the sigmoid notch. Its anterior border contained an undulating recess serving as docking site for the second PSD component, i.e. a “tandem” washer for load distribution and reinforcement of the LSFPSO.

The upper PSD portion included six screw holes arranged in two rows (four along the common arch brace and one on each of the two central dome extensions) to ensure adequate fixation of the reassembled fragments, even if not every screw hole was occupied. The lower PSD portion contained three screw holes in a triangular configuration for anchoring to the condylar neck and base region.

The PSD “tandem” washer seated within the lateral recess along the first PSD component. This second PSD combined washer and navigation function: the tongue-in-groove design of the two components ensured accurate intraoperative transfer of the LSFPSO entry points onto the lateral fracture end at the ramus. Besides the two washer screw holes the PSD contained a third smaller hole for its temporary fixation during LSFPSO.

Detachable drill guides, designed to indicate the angulation and intramedullary pathways for the LSFPSO screws within the medial fragment assembly, were provided in two versions: two separate metallic cylinders joined by a polymer sleeve with and without a long rod-like handle.

The PSD tool set was complemented with a third component, a small T-shaped implant to augment the rigidity of the overall reconstruction after removal of the primary (reduction/prefixation) PSD. The T-shaped PSD was conceived to link the condylar neck and head and retain the small intermediate dorsal monocortical fragment (SIDMF) at the posterior condylar surface.

### Surgical Procedure

The dislocated major dome fragment, along with the smaller medial fragments, were successfully reassembled and anatomically reduced. However, repeated attempts to position the reduction/prefixation PSD component as pre-planned failed. Unresolvable collisions between the dome fragment, the cranial PSD extensions and the glenoid fossa hindered the first component from bypassing the bony crest and slipping into its planned final position.

For salvage the large medial condylar head fragment was reduced and fixated to the lateral fracture end with a conventional grid plate (1.2 mm screws; Medartis). To achieve this, it became necessary to remove the SIDMF and to close the interfragmentary gap by a slight pitch movement. This adjustment was required because the available grid plate was too short.

The entry points for the LSFPSO were established with the aid of the washer PSD. Despite the discrepancies from VSP, the placement of the washer components, drill guides and drilling could be carried out with sufficient precision. Because the bone surrounding the drill holes did not necessitate load distribution the washer PSD was removed prior to insertion of two screws (1.8 mm screws; Medartis) for the LSFPSO.

Occlusion and latero-/protrusion movements were assessed intraoperatively and confirmed satisfactory fracture reduction and stabilisation. Consequently, the grid plate was removed. Minimisation of the residual gap and SIDMF replantation were deemed unnecessary. Further stabilization with the T-shaped PSD was also not pursued.

### Outcome

A postoperative panoramic X-ray confirmed anatomical reduction of the fracture.

Clinical Follow up: asymptomatic with unimpaired function.

## Discussion

LSFPSO using two or three screws has emerged as the most popular surgical treatment modality for CHF [2, 6]. The concept of combining PSDs with screw fixation might offer a promising approach for treating challenging CHF up to most troublesome multifragment patterns [13].

Pavlychuk et al. have outlined sophisticated CAD/CAM PSD designs to support ORIF of CHFs across three publications [13–15]. For unclear reasons, their concept has not met much resonance in the literature so far. Our case series showcases the intraoperative constraints encountered with three single-component and one multi-component PSD, the latter inspired by Pavlychuk et al.

### Roles and Advantages of PSD in CHFs

PSDs are used alongside LSFPSO. PSDs can be tailored to perform multiple functions, including:

- Assist reduction, reassembly and prefixation of medial condylar head fragments.
- Navigate LSFPSO entry points.
- Guide drilling for intramedullary screw placement (LSFPSO).
- Augment osteosynthesis rigidity through reinforcement.

PSDs may be employed intraoperatively only or retained for long-term stability. Their advantages over off-the-shelf hardware include improved fragment reduction accuracy, enhanced rigidity, and reduced operative time, as evidenced in other surgical contexts [10, 11].

### Insights from this Case Series

A principal cause for the pitfalls described above was limited experience with nuanced PSD design features for CHF management. Crucially, salvage solutions were at hand in all cases. To flatten the learning curve in order to establish and help to disseminate PSDs in surgical CHF treatment, we would like to refer to the following recommendations and caveats for the PSD design:

- VSP–Fracture Line Analysis: Accurate analysis of the fracture line and reduction of fragments in the planning phase is essential – rather than simply relying on mirroring and superimposition of intact contralateral structures. Mandibular asymmetry will compromise the true morphology and course of fracture lines [13].
- Intraoperative Visualization: Ensure a reliable overview of the entire fracture line course during every phase of the surgical intervention by incorporating large fenestrations or cross-bracings into the PSD design. This principle has been repeatedly exemplified in wire-frame patient-specific cutting guides [10, 11].
- Flexible Strategies for Screw Fixation: Provide an excess of drilling/ screw−receiving holes in the PSD design, even if only the most suitable subset of the surplus of screw holes may be occupied to deal with unforeseen problems, such as poor bone quality or inaccessible anatomic sites. The integration of screw holes in excess numbers into osteosynthesis plates designs to increase intraoperative flexibility has already been described in context with condylar base and neck fractures [14].
- Thoughtfully executed Coverage across Relevant Anatomical Sites: Extend the PSD to encompass the infero−lateral surface of the medial condylar head fragment approaching to the margin of the fracture line. This is an easily accessible area, which facilitates screw insertion besides control of accurate reduction of the inferior part of the fracture.
- Temporary Use of Grid Plates: Remove grid plates or medial fragment−reduction PSD component after LSFPSO is secured unless a “stand−by” stabilization is required.

Loosening of positional screws and conventional grid plates is a known issue in ORIF of CHF [2]. Possible causes include poor bone quality or cyclic loading, leading to the backout of screws [15]. Therefore, a grid plate or PSD should be removed subsequent to the LSFPSO to preclude a secondary hardware removal. An exception is the need for additional stabilization via “standby” with extrabony fixation devices.

### Case-specific Insights and Recommendations

At the treatment time of Case 4, we have been overly enthusiastic in adopting ideas from the novel PSD design concept that had just been introduced by Pavlychuk et al.[13–15]. Accordingly several PSD components with different purposes were tailored to work in a sequentially coordinated fashion. A primary reduction/prefixation PSD component was created to catch and stabilize a multitude of medial condylar head fragments within a cage−like framework. The cranial end of this cage carried four terminal phalanges reaching over the domal crest of the reassembled fragments. Although the PSD could, by and large, be accommodated within the fracture site, we failed to advance the extensions across the dome convexity. All attempts led to unresolvable dislodgments and made the application of the following PSD components impossible.

As a consequence of this misassessment that had its origin in the VSP/CAD phase, we recommend to omit extensions, overhangings or undercuts within morphological transition zones, in order to prevent intraoperative hardware collisions.

Similar design flaws with increasingly complex PSD have been observed in other settings, e.g., bilateral sagittal split ramus osteotomies with CAD/CAM technology [16].

Our motive to apply PSDs in CHF surgical treatment was the search for simplification and improvement of accuracy. Currently their benefit is not yet sufficiently ascertained; there is a clear need to identify refined indication criteria particularly in multifragmentation fractures. In cases of extremely damaged joints, total joint replacement (TJR) may be considered alternately. While traditionally reserved for end-stage TMJ-pathology, Pruszyńska et al. suggest TJR as primary option for multifragmented CHF if the mandibular ramus height cannot be reliably restored via ORIF [17, 18].

**Table 1:** The comprehensive AO−CMF−classification−system for mandibular condylar head fractures.12

Region	Fracture Location	Fragmentation	Displacement
Condylar Head	m = medical to the lateral pole zone	0 = none	0 = complete vertical apposition/ fragment contact
	p = within or lateral to the lateral pole zone	1 = minor	1 = partial vertical apposition/fragment contact
		2 = major	2 = lost vertical apposition/fragment contact
